# The roles and applications of short-chain fatty acids derived from microbial fermentation of dietary fibers in human cancer

**DOI:** 10.3389/fnut.2023.1243390

**Published:** 2023-08-08

**Authors:** Yuanqing Li, Yaxuan Huang, Haili Liang, Wen Wang, Bo Li, Ting Liu, Yuqi Huang, Zhe Zhang, Yutao Qin, Xiaoying Zhou, Rensheng Wang, Tingting Huang

**Affiliations:** ^1^Department of Radiation Oncology, First Affiliated Hospital of Guangxi Medical University, Nanning, China; ^2^Key Laboratory of Early Prevention and Treatment for Regional High Frequency Tumor (Guangxi Medical University), Ministry of Education, Nanning, China; ^3^Department of Otolaryngology-Head and Neck Surgery, First Affiliated Hospital of Guangxi Medical University, Nanning, China; ^4^Guangxi Zhuang Autonomous Region Institute of Product Quality Inspection (GXQT), Nanning, China; ^5^The First School of Clinical Medicine, Guangxi Medical University, Nanning, China; ^6^Life Science Institute, Guangxi Medical University, Nanning, China

**Keywords:** dietary fiber, gut microbiota, short-chain fatty acids, cancer, immunotherapy

## Abstract

Dietary fibers (DFs) and their metabolites attract significant attention in research on health and disease, attributing to their effects on regulating metabolism, proliferation, inflammation, and immunity. When fermented by gut microbiota, DFs mainly produce short-chain fatty acids (SCFAs), such as acetic acid, propionic acid, and butyric acid. As the essential nutrients for intestinal epithelial cells, SCFAs maintain intestinal homeostasis and play essential roles in a wide range of biological functions. SCFAs have been found to inhibit histone deacetylase, activate G protein-coupled receptors, and modulate the immune response, which impacts cancer and anti-cancer treatment. Notably, while extensive studies have illuminated the roles of SCFAs in colorectal cancer development, progression, and treatment outcomes, limited evidence is available for other types of cancers. This restricts our understanding of the complex mechanisms and clinical applications of SCFAs in tumors outside the intestinal tract. In this study, we provide a comprehensive summary of the latest evidence on the roles and mechanisms of SCFAs, with a focus on butyric acid and propionic acid, derived from microbial fermentation of DFs in cancer. Additionally, we recapitulate the clinical applications of SCFAs in cancer treatments and offer our perspectives on the challenges, limitations, and prospects of utilizing SCFAs in cancer research and therapy.

## Introduction

Recent research has highlighted the significant impact of dietary fibers (DFs) on human health (1, 2) influencing the risk of chronic diseases, such as cancer, obesity, type 2 diabetes, and cardiovascular diseases (3, 4). DFs encompass soluble and insoluble fibers, which are a group of carbohydrates that cannot be digested or absorbed in the small intestine (3, 5). Soluble fibers, including oligo galactose, oligofructose, inulin, β-glucan, resistant starch, and pectin, are widely recognized as prebiotics (6). When fermented by gut microbiota, soluble fibers mainly produce short-chain fatty acids (SCFAs), such as acetic acid, propionic acid, and butyric acid (7).

SCFAs, which serve as essential nutrients for colonocytes and gut microbes, play a crucial role in maintaining intestinal and systemic homeostasis, impacting lipid and glucose metabolism, cell proliferation, inflammation, and immune system functionality (7, 8). In particular, the roles of butyric acid and propionic acid have been extensively investigated, revealing their contributions to health and diseases, including human cancers. It is suggested that butyric acid and propionic acid act as histone deacetylase inhibitors (HDACIs) to epigenetic modulate gene expression, influencing cell growth, proliferation, and apoptosis (9–12); act as ligands for G protein-coupled receptors (GPCRs), regulating cell proliferation, apoptosis, and immune response (11, 13); furthermore, they exhibit anti-inflammatory and immunomodulatory effects by regulating inflammatory factors and cytokines and promoting the differentiation and migration of immune cells (10, 11, 14–16).

Notably, while extensive studies have illuminated the roles and applications of SCFAs in colorectal cancer (CRC) (17–22), limited evidence is available for other types of cancers. This restricts our understanding of the roles of SCFAs in tumors outside the intestinal tract and the complex mechanisms underlying the regulation of the tumor-immune microenvironment (TIME). In this study, we provide a comprehensive summary of the latest evidence on the roles and mechanisms of SCFAs, with a focus on butyric acid and propionic acid, derived from microbial fermentation of DFs in cancer. Additionally, we recapitulate the clinical applications of SCFAs in cancer treatments and offer our perspectives on the challenges, limitations, and prospects for utilizing SCFAs in cancer research and therapy.

## The roles and mechanisms of SCFAs in cancer

### Functioning as epigenetic modificators

SCFAs as HDACIs play a crucial role in the epigenetic regulation of gene expression, influencing cell survival, proliferation, and differentiation (23, 24). Numerous *in vitro* studies have demonstrated that SCFAs presented HDACI activities in various cancer cell lines, including (9, 25–27) breast (28), gastric (29), and cervical cancer (30). SCFAs have been shown to inhibit cell proliferation, induce cell cycle arrest at G0/G1 or G2/M phase, trigger apoptosis mediated *via* the mitochondrial pathway, promote autophagy, and increase the accumulation of reactive oxygen species (ROS). In a study of BALB/c nude mouse model with HCT-116 cells inoculation by Ma et al. (31), sitosterols feeding elevated diversity of gut microbiota, increased levels of SCFAs in fecal samples, and restrained CRC cell growth. The study further revealed that SCFAs induced tumor apoptosis through the PI3K/Akt pathway and altered the expression levels of apoptosis-related proteins, such as Bad, Bcl-xl, and cytochrome C (31). Hence, SCFAs by acting as HDACI show potential as attractive targets for developing novel therapeutic strategies, as discussed in Section 3.

### Acting as G protein-coupled receptor ligands

SCFAs are natural ligands for the G protein-coupled receptors (GPCRs), including GPR43 (also termed free fatty acid receptor, FFAR2), GPR41 (also termed FFAR3), and GPR109A (13, 32). In colon cancer cells, by combining these receptors, SCFAs inhibit cell proliferation, induce apoptosis, and cycle arrest *via* the NF-κB, MAPK, ERK1/2, PI3K, and Wnt signaling pathways (13, 32). For instance, SCFAs induced cell proliferation inhibition, apoptosis, and invasion inhibition, mediated by GPR43 in colon cancer cells (9, 33), HeLa cells (34), BaF3 leukemia cells (35), and breast cancer cells (36). Propionate and butyrate are high-affinity ligands for GPR43, dual-coupled to the pertussis-sensitive Gαi/o and Gq protein, and reduce cAMP levels (37). Similarly, Yonezawa found that both GPR41 and GPR43 were expressed in breast cancer cell lines; while combining with SCFAs, they raised intracellular concentration of Ca^2+^ and activated the p38 MAPK pathway, thereby inhibiting cell proliferation (38) (Table 1). In an intestinal cancer model, Kim et al. (40) observed that the SCFA-GPR43 axis suppresses the Th17-driven inflammatory response and intestinal carcinogenesis. In addition, GPR109A binds only to butyrate and reduces cAMP through Gαi/o proteins (37). GPR109A mediated butyrate anti-cancer activity in colon cancer cell lines by inhibiting the activation of NF-kB, downregulating anti-apoptotic genes, and upregulating pro-apoptotic genes (9, 41). Moreover, propionate and butyrate could activate GPR41 which was coupled through Gαi/o proteins to reduce cAMP (37), increase the intracellular concentration of Ca^2+^, and inhibit the MAPK signaling pathway to lower the invasion of breast cancer cells (36).

**Table 1:** SCFA receptors and related signaling pathways.

**GPCR**	**Ligands**	**Tissue/cell expression**	**Signaling pathways**	**References**
GPR41 (FFAR3)	Propionate butyrate	Adipose tissue, colon, spleen, lymph nodes, and bone marrow	Increase histone acetylation and involve in the regulation of acetylation-related cellular processes; reduce cAMP through Gαi/o; increase in intracellular Ca^2+^; inhibit MAPK signaling pathway	(32, 36, 38, 39)
GPR43 (FFAR2)	Propionate butyrate	Immune cells, neutrophils, monocytes, gastrointestinal epithelial cells, adipocytes, enterocytes, and endocrine	Reduce cAMP through Gαi/o and Gαq proteins; p38 MAPK/HSP27 pathway; inhibit the Hippo-Yap pathway and increase E-cadherin to inhibit invasion; ↓*Bcl-2*, ↓*Survivin*, ↓*cyclinD1/D3*, ↓*CDK1*, ↓*CDK21*, ↓*PCNA*, ↑*p21*, activate caspases-3/6/7/8, G0/G1 cell cycle arrest; suppress Th17-driven inflammatory response and intestinal carcinogenesis	(33, 34, 36, 38, 40)
GPR109A (HCAR2)	Butyrate	Adipocytes, immune cells (neutrophils, dendritic cells, and macrophages), retina, and colon	Reduce cAMP through Gαi/o proteins; ↓*Bcl-2*, ↓*Bcl-W*, ↓*Bcl-xL*, ↓*Bfl-1*, ↓*cyclin D1*, ↑*FAS-L*, ↑*FAS-R*, ↑*FADD*, ↑*TNF-R1*, ↑*PTEN*, ↑*PPARγ*, ↑*Foxo3A*, inhibit NF-κB; activate caspase-3/8/9	(32, 41)

### Regulating TIME

**Figure 1:**
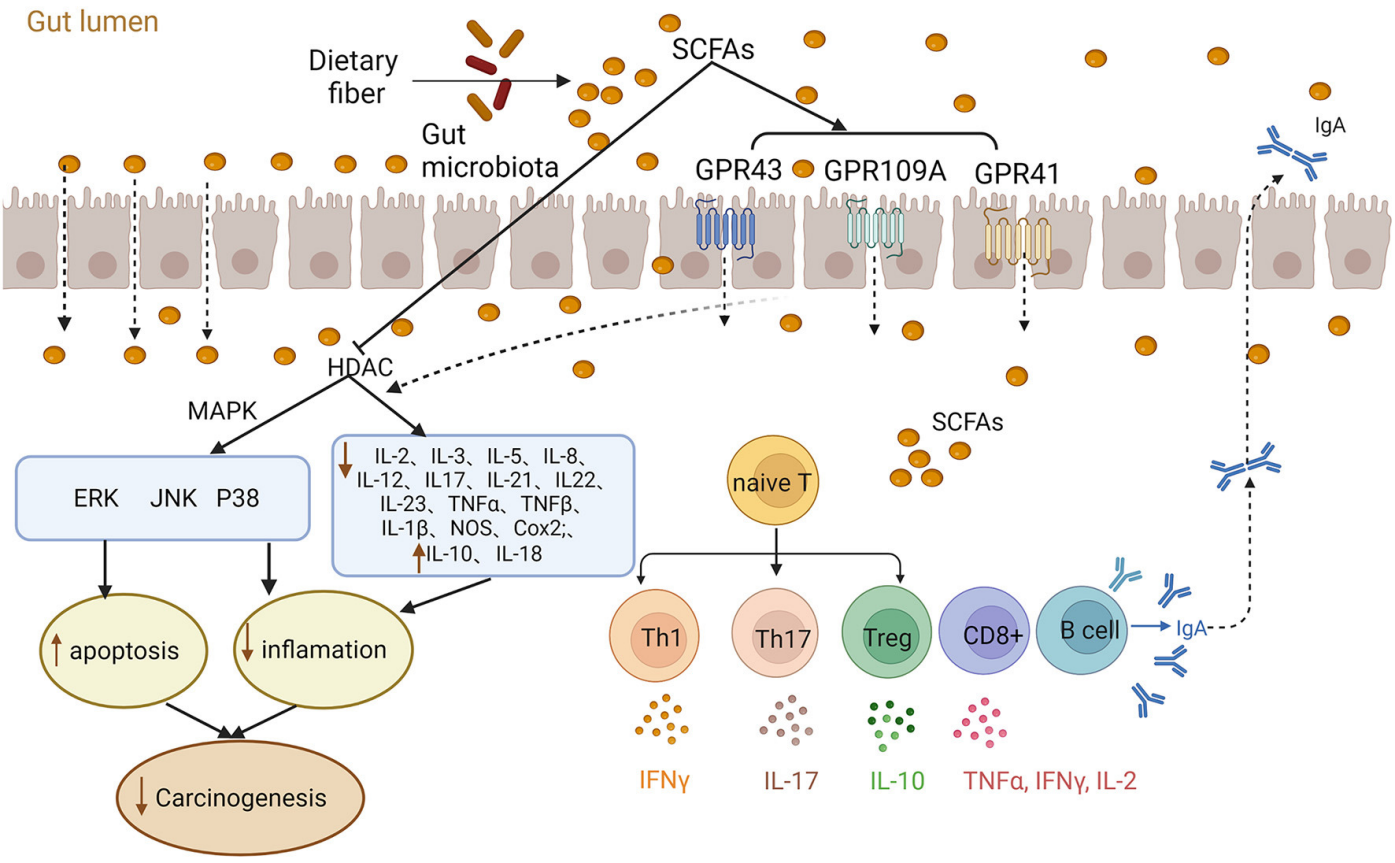
Role of short-chain fatty acids (SCFAs) in inflammation and immunity. The gut microbiota ferments dietary fibers producing SCFA that exert their effects through various mechanisms. SCFAs can act as histone deacetylase inhibitors (HDACIs) and activate G protein-coupled receptors (GPCRs) to modulate cellular responses. For instance, SCFAs regulate MAPK signaling pathways (ERK, JNK, and p38) and stimulate the release of inflammatory factors, contributing to inflammation and carcinogenesis. Additionally, SCFAs play a vital role in immune regulation by promoting the differentiation of naive T cells into effector cells and regulatory T cells (Tregs). Moreover, SCFAs influence cytokines release, impacting intestinal homeostasis and immunity.

SCFAs play essential roles in the host immune system, such as influencing the differentiation of myeloid and lymphocytes (42–44). SCFAs exert their immunomodulatory effects through two primary mechanisms: acting as HDACIs and interacting with GPCRs (43) (Figure 1). For example, *in vitro* and *in vivo* investigations involving C57BL/6 mice, various gene-deficient mouse models [*Rag1(–/–), GPR41(–/–), GPR43(–/–), IL-10(–/–)*] and T cell lines (CD4 ^+^, CD8 ^+^) showed that SCFAs promoted the differentiation of naive T cells into effector cells (Th1 or Th17) or regulatory T cells (Tregs). This regulation influences the production of IL-17, IFN-γ, and IL-10, thereby affecting immunity or immune tolerance (45). Additionally, SCFAs regulate the MAPK signaling pathways (ERK, JNK, and p38) to modulate immune and endothelial cells, leading to the suppression of inflammation and tumors (16). SCFAs have been observed to suppress inflammatory cytokines IL-1β, IL-2, IL-3, IL-5, IL-6, IL-8, IL-12, IL-17, IL-21, IL-23, TNF-α, TNF-β, NOS, and COX2, while increasing the expression of anti-inflammatory cytokines IL-10 and IL-18. This reduction in inflammation contributes to the suppression of CRC development (9, 11, 16, 22, 32). Furthermore, SCFAs affected both innate and adaptive immune responses by stimulating B cells to secrete intestinal immunoglobulin A (IgA) (13, 46, 47). Notably, a study by Luu et al. (48) demonstrated that SCFAs enhanced the secretion of cytokines (including IL-2, TNF-α, and IFN-γ) by modulating CD8^+^ T cells, thereby improving cancer immunotherapy.

### Carcinogenic effects of SCFAs

While SCFAs have commonly been recognized as tumor-suppressive metabolites, it is noteworthy that under certain conditions, SCFAs can promote tumorigenesis (49–51). Matsushita et al. (52) conducted research using prostate-specific *Pten* knockout mice (*Pb-Creþ; Pten*^*fl*/*fl*^) and prostate cancer cell lines (DU145, 22Rv1) to demonstrate that SCFAs supplementation promoted prostate carcinogenesis by increasing insulin-like growth factor-1 production. Another study reported that long-term consumption of fiber-enriched foods in dysbiosis mice resulted in hepatocellular carcinoma (HCC) (53). In addition, a mouse model with colon cancer driven by mutations in the mismatch repair gene *Msh2* and *Apc* gene showed that butyrate promoted the development of CRC (54). Okumura et al. (55) have currently described that the overgrowth of *Porphyromonas* species in an *Apc*^Δ14/+^ mouse model is casually related to colorectal cancer due to butyrate-engaged senescence. Notably, scientists have long debated these opposing observations and dubbed the phenomenon “butyrate paradox” (51). Given the “Warburg effect” (56), it has been widely accepted that butyrate provides energy to normal cells to promote cell growth. In contrast, cancerous cells instead relied on aerobic glycolysis; therefore, butyrate accumulated and functioned as an HDACI to halt cell cycle progression. Surprisingly, growing evidence in colon cancer cells showed that butyrate could directly combine and change the metabolic enzymes, leading to an anti-tumor effect without following the “Warburg effect” (57–59). Moreover, GPR41 could decrease butyrate-induced histone acetylation and negatively regulate butyric-induced anti-proliferative and apoptosis (39). Thus, it would be narrow to define butyrate or SCFAs simply as onco-metabolites or tumor-suppressive metabolites, given their complex effects that are waiting for exploration.

## Advancements of gut microbiota-derived SCFAs in cancer treatment

SCFAs gained attention in the 1980s when butyrate was reported to modulate the malignant biological behavior of cultured colon cancer cells (60, 61). Sodium butyrate has been shown to inhibit the growth of hepatocellular carcinoma (HCC) cells in both *in vitro* using the HuH-7 human HCC cell line and *in vivo* utilizing an HCC tumor-bearing mice model (62). These inhibitory effects are likely mediated by a p21-dependent mechanism. In addition, sodium butyrate has demonstrated the ability to hinder the G1-S transition of human glioma cells, as evidenced by increased expression of p21 and cyclin D1, and reduced phosphorylation of pRb (63). It has also been found to impede cell proliferation in the MCF-7 human breast cancer cell line, reflected by increased expression levels of p21WAF1 and RARβ (64). Moreover, sodium butyrate induced AMPK-mTOR-mediated autophagy and ROS-mediated apoptosis of bladder cancer cells (T24, 5637, and SV-HUC-1 bladder cancer cell lines) (65), induced DAPK-mediated apoptosis in human gastric cancer cell lines (AGS, Kato III, etc.) (66), and triggered mitochondrial-mediated apoptosis in colon cancer cell line (Caco-2 cell line) (67). However, the translation of SCFAs to clinical applications has been impeded by their low concentration in peripheral blood and rapid plasma clearance (68), which will be further discussed in Section 4. Two decades later, with the iteration of sequencing technology, the association between gut microbiota-derived SCFAs and their role as anti-cancer agents once again captured scientists' attention for SCFAs as anti-cancer agents.

The investigations of the association between SCFAs and cancers fell into several research modes as follows:

1) ***In vitro*** **studies**. Nakkarach et al. (69) isolated the bacterial strain (*Escherichia coli* KUB-36) from fecal samples collected from healthy individuals which demonstrated the highest production of SCFAs. The researchers applied the metabolites and individual SCFA to various tumor cell lines, including breast cancer, colorectal cancer, and leukemia. Remarkably, all treatments exhibited inhibitory effects on tumor cell growth, with breast cancer cells showing the greatest sensitivity to the treatments (69). Additionally, Zheng et al. indicated that secretions of *C. butyricum* induced cytotoxic effects on CRC cells, including human CRC cell lines HCT116 and HT29, as well as the mouse CRC cell line CT26. However, the subsequent addition of butyrate kinase inhibitors impaired the cytotoxic effects specifically in CT26 cells, providing strong evidence that the anti-cancer effect of *C. butyricum* was mainly attributed to the secretion of butyrate (70).
2) ***In vivo*** **studies**. In a recent study, it was demonstrated that the concentration of intestinal SCFAs concentration in mice with HCC can be increased by administering a probiotic mixture named Prohep. Prohep, composed of Lactobacillus rhamnosus GG, *Escherichia coli* Nissle 1917, and VSL#3, was found to confer tumor suppression effect. This effect was associated with alterations in the composition and diversity of gut microbiota and an increase in SCFA-producing bacteria in the group of mice treated with the probiotic mixture (71). The intervention with Prohep appeared to be relevant to the downregulation of IL-17, the reduction of Th17 polarization, and the differentiation of Treg/Tr1 (72). In another study, the effect of SCFAs on extra-intestinal tumor progression was investigated in a mouse model of lung metastasis from melanoma. Supplementation with VSL#3, a registered probiotic formula consisting of eight different strains of probiotic bacteria, resulted in an increased amount of propionate and butyrate in plasma and fecal samples. Subsequent analysis showed that these SCFAs significantly decreased the volume of tumors, possibly by recruiting Th17 cells to the lung tissue through the chemokine ligand 20/chemokine receptor 6 axis (73).
3) **Multi-omics analysis**. Multi-omics analyses have emerged as novel approaches, integrating metagenomic, transcriptomic, proteomic, metabolomic, and lipidomic analysis. These comprehensive investigations shed further light on the host's response to probiotics at multiple levels (74). For instance, in a mouse model of HCC treated with probiotics, researchers utilized metagenomic analysis to identify altered pathways and corresponding biological functions (71). Notably, they observed significant changes in pathways involved in SCFAs synthesis within tumor cells. Furthermore, applying metabolomic analysis provides valuable insights into the modulation of metabolite profiles following probiotic intervention (75). In a study that combined metagenomics and metabolomics (using gas chromatography-mass spectrometry, GC-MS), researchers screened for phages associated with CRC promotion (mainly *Fusobacterium nucleatum*) and inhibition (mainly *Clostridium butyricum*). Through gene ontology enrichment analysis, differentially expressed genes were found to be enriched in apoptosis and autophagy, uncovering the potential mechanism. Additionally, GC-MS analysis of *C. butyricum's* secretome revealed that butyrate played a prominent role in the cytotoxic effects on CRC cells (70).

While many studies regarding SCFAs in cancer management provided valuable insights into their potential effects and mechanisms, these preclinical studies were limited *in vitro* and *in vivo*. It is essential to conduct well-designed clinical trials (including double-blinded or triple-blinded studies) to further investigate the efficacy and safety of SCFAs in human subjects.

### SCFAs combined with chemotherapy and radiotherapy

Recently, SCFAs have been studied as a sensitizer for radiotherapy and chemotherapy. Sodium butyrate combined with cisplatin has been described to promote apoptosis in different tumor cells, such as gastric cancer (76) and cervical cancer (77) *in vitro* and *in vivo*. In the tumor-bearing mouse model of gastric cancer, butyrate plus cisplatin inhibited tumor growth *via* the mitochondrial apoptosis-related pathway, surpassing other groups with monotherapy (76). The combination of butyrate and cisplatin has been reported in the cervical cancer model (Hela and Siha cell lines and tumor cell-inoculation mice) that inhibited cell migration and invasion by blocking the nuclear conversion of β-catenin, reversing epithelial–mesenchymal transition, upregulating the expression of E-cadherin and downregulating matrix metalloproteinase (MMP)2, MMP7, and MMP9 (77). In addition, Park et al. (78) investigated the effects of radiotherapy combined with butyrate, propionate, and acetate in organoids. Among them, butyrate showed radio-sensibilization and weak toxicity to normal mucosa and inhibited the proliferation of organoids. Data on the safety and efficacy of the combination in animal studies and clinical trials are yet to come.

### SCFAs combined with immunotherapy

Several studies focused on patients with different types of cancer receiving immune checkpoint inhibition (ICI) therapy and collected patients' fecal samples (20, 79, 80). They suggested that the concentration of SCFAs in fecal samples might be associated with the efficacy of anti-programmed cell death protein 1 (PD-1) and anti-programmed death-ligand 1 (PD-L1) immunotherapy. These findings prompt that gut microbiota links to ICI therapeutic efficacy through SCFAs, which show the potential to be a response marker. Animal studies found that SCFAs had diverse effects on different ICI therapies (81, 82). In a CRC mouse model, researchers found that the dietary supplement of pectin increased butyrate production in the gut, promoted T-cell infiltration, and enhanced the anti-cancer effect of anti-PD-1 drugs in CRC mice (81). Another mouse model CRC/fibrosarcoma reported that butyrate restrained anti-CTLA-4 response through downregulating CD80/CD86 on dendritic cells and Inducible costimulatory on T cells and preventing the accumulation of tumor-specific T cells, memory T cells, and IL-2 (82).

### SCFAs in the comprehensive management of cancer

SCFAs have therapeutic potential in treating intestinal inflammation induced by chemotherapy or radiotherapy. They reconstruct the intestinal epithelium barrier and regulate intestinal immunomodulatory function (83). In addition, direct administration of SCFA-producing bacteria (probiotics) can restore intestinal ecology and inhibit the secretion of proinflammatory cytokines (84). In the perioperative management of resectable tumors, the application of SCFA-producing bacteria (probiotics) could decrease the incidence of postoperative complications (85, 86). For CRC patients, adding butyrate before the operation helps to improve the integrity of the intestinal barrier (87).

### SCFAs from dietary fibers supplementation in cancer treatment

Numerous studies support the health-promoting effects of DFs from daily food (88, 89), including the anti-tumor effect. Pectin and inulin have been reported to enhance the immune response to tumors in mouse models. Pectin supplementation was associated with an improved response to immunotherapy in mice with CRC (81). Another study suggested a potential link between SCFAs derived from inulin fermentation and the anti-tumor activity of ICIs (90). Nevertheless, pectin has been shown to accelerate carcinogenesis in *Apc*-deficient mice (91), while dietary inulin supplementation may induce gut microbiota-dependent hepatocellular carcinoma (53). In addition to animal experiments, clinical research has indicated that adequate DFs intake can improve the prognosis of cancer patients. A cross-section study revealed that sufficient DFs intake was associated with significantly improved PFS and response to ICIs in melanoma patients, compared to a combination of DFs and probiotics (92). However, the study did not find a significant association between DF proportions and the SCFA levels in the gut. Furthermore, SCFAs play a critical role in the health-promoting effect of vegetarian and Mediterranean diets, which are characterized by high DF content (93–95). Nevertheless, the absence of relevant cohort studies makes it uncertain whether cancer patients can benefit from these dietary patterns. These findings highlight the need to carefully evaluate the potential benefits of DFs in future studies, considering their potential risks.

## Challenges and limitations

### Challenges as a therapeutic approach for cancer

The anti-cancer drug usually requires a comprehensive understanding of its pharmacology, toxicology, and high specificity on its target molecules. SCFAs have been found ambiguity effects on tumor progression: suppression and promotion, which challenges the further application of SCFAs in anti-cancer treatment. Donohoe et al. (96) reported decreased production of butyrate and increased butyrate nuclear accumulation in a microbiota- and butyrate-dependent mouse model with colon tumor cells. These phenomena were associated with enhanced apoptosis and reduced proliferation in tumors. Another mouse model with colon cancer driven by mutations in the mismatch repair genes *Msh2* and *Apc* showed that butyrate drove the hyperproliferation of *Msh2*-deficient epithelial cells and promoted the development of CRC (54). Noteworthy, tumor genetics and butyrate concentrations were considered the key factors that led to the opposite effects of SCFAs on carcinogenesis between these investigations mentioned above (49, 50). So far, the questions about which are the responsible mutations and what is the cut-off concentration still need to be answered. It indicates that researchers should be aware that SCFAs may play more complex and comprehensive roles in cancer than we used to understand. Thus, we urge that more efforts be put into unraveling the spectrum of SCFAs' biological effects on cancer.

### Limitations of distribution and plasma clearance

SCFAs serve as the primary energy source for intestinal epithelial cells; therefore the systemic absorption of butyrate is low (51). Their concentrations significantly differ between enteral and abenteric environments (butyrate concentration is 29 μM in portal vein vs. 4 μM in peripheral circulation) (68, 97). To engage their anti-tumor effects, SCFAs shall maintain different effective concentrations continuously in a patient's circulation given cancer types. For example, butyrate concentration in circulation should reach at least 0.5 mM to induce tumor cell differentiation in CRC (98) and breast cancer (28), However, butyrate at the concentration of 0.5 mM did not significantly affect the gastric cancer cell viability *in vitro* experiments (76). In addition, butyrate has a rapid plasma clearance in the human body with only a 6 min half-life. Once absorbed, SCFAs are transported to the liver *via* portal circulation and become the substrate for longer-chain fatty acids (51). Researchers reported that the peak concentration of butyrate in plasma among patients with acute leukemia was merely 0.05 mM by intravenous infusion (99). The insufficient concentration and short half-life of SCFAs in human circulation challenge their application. Current efforts have been made to innovate drug administration and explore stable derivatives:

1) **Drug administration**. Oral administration of solid lipid nanoparticles (SLN) (100) is an attempt to deliver butyrate across the intestinal barrier to target organs using a sustained-release drug delivery system. SLN is not absorbed by the gastrointestinal tract and cannot pass through the blood–brain barrier. Cholesteryl-butyrate SLN has been confirmed to increase the stability and efficacy of butyrate in a mouse glioma model (100).
2) **Stable derivatives**. Researchers tried to use prodrugs of SCFAs [Trybutirin (101), phenylbutyrate (102), and pivaloyloxymethyl butyrate (Pivanex, AN-9) (103, 104)] and explore their effects on tumors [leukemia (102), non-small cell lung cancer (104), and prostate cancer (105)]. These prodrugs had not only similar effects as butyrate in inducing apoptosis (101) and anti-angiogenesis effects (106) but also longer half-life and higher stable plasma concentrations (107). Notably, the doses were still insufficient to exert consistent anti-tumor effects (108).

To sum up, exploring various local delivery methods (such as enema, nasal spray, aerosol inhalation, intravaginal administration, and bladder irrigation) or developing new drug delivery systems may be the direction of future translational research.

## Conclusion and future perspectives

Although astounding clinical successes in anti-cancer treatments have been achieved, cancer remains the second leading cause of death worldwide and dramatically affects the quality of life of cancer survivors. In the present review, we summarize advancements in the roles of the microbial fermentation of DFs-derived SCFAs in cancer and recapitulate the up-to-date evidence on the applications of SCFAs in cancer treatment. Additionally, we notice that SCFAs present the potential to mediate a wide range of biological effects beyond function as HDACIs, GPCRs, and TIME modulators, resulting in both tumor suppression and promotion. It highlighted the challenges of applying prebiotics, probiotics, and microbial metabolites to a therapeutic modality for cancer. We urge more effort to be put into unraveling the spectrum of SCFAs' biological effects and their functional organizing network, which is the prerequisite for better management of cancer.

Moreover, SCFAs might influence carcinogenesis and inflammation similarly in other regions beyond the gut, such as the reproductive tract, respiratory tract, and urinary tract. A fiber-rich diet can increase the production of SCFAs by altering the composition, diversity, and abundance of the microbiome to promote health. Hence, we might regulate SCFAs by prebiotics or probiotics to alter the commensal microbiome and modulate the desirable concentration of SCFAs in particular regions. To test these hypotheses, future investigations are warranted to explore the associations between commensal microbiota and its metabolites in various body sites and various types of cancer, consequently developing novel therapeutic approaches for improving prognosis and quality of life among cancer patients.

## Author contributions

All authors listed have made a substantial, direct, and intellectual contribution to the work and approved it for publication.
